# TAFRO syndrome complicating Sjögren’s disease: response to subcutaneous tocilizumab

**DOI:** 10.1007/s10067-026-08075-1

**Published:** 2026-05-23

**Authors:** N. A. Uribe-Ruíz, N. Murillo–Baquero, A. F. Vargas-Camacho, V. Santiago-Pacheco, A. Taborda-Murillo, C. H. Muñoz-Vahos, L. A. González-Naranjo, A. L. Vanegas-García

**Affiliations:** 1 Grupo de Reumatología, Departamento de Medicina Interna, Universidad de Antioquia, Carrera 129 # 61-38, Interior 201, Código Postal: 050036, Medellín, Colombia; 2 Departamento de Medicina Interna, Universidad de Antioquia, Medellín, Colombia; 3 Departamento Patología, Universidad de Antioquia, Medellín, Colombia; 4 Hospital Universitario San Vicente Fundación, Medellín, Colombia; 5 Comité de Estudios Médicos, Medellín, Colombia; 6 Asociación Colombiana de Reumatología, Medellín, Colombia

**Keywords:** IL-6, Sjögren’s disease, Systemic inflammatory response, TAFRO syndrome, Tocilizumab

## Abstract

TAFRO syndrome is a rare hyperinflammatory disorder characterized by thrombocytopenia, anasarca, fever, reticulin fibrosis, renal dysfunction, and organomegaly. Its coexistence with Sjögren’s disease has only been reported in isolated cases, and both conditions are thought to share a pathogenic pathway mediated by the activation of the IL-6/VEGF axis and the presence of anti-SSA/Ro60 autoantibodies. We present a 33-year-old woman with primary Sjögren’s disease who fulfilled the 2016 ACR/EULAR criteria and developed anasarca, persistent fever, severe thrombocytopenia, and acute kidney injury due to thrombotic microangiopathy and tubulointerstitial nephritis. Despite receiving pulse therapy and high-dose glucocorticoids, she experienced progressive deterioration that required renal replacement therapy. Given the suspicion of TAFRO syndrome associated with a systemic inflammatory response, weekly subcutaneous tocilizumab was initiated, resulting in rapid improvement in her overall condition, resolution of anasarca, and complete recovery of renal function. This case highlights the rare overlap between TAFRO syndrome and Sjögren's disease, in which IL-6 activation plays a central role. The favorable response to IL-6 blockade emphasizes the importance of early recognition of this association and consideration of targeted therapies for refractory hyperinflammation.

## Introduction

Sjögren’s disease (SjD) is a systemic autoimmune disorder characterized by lymphocytic infiltration of the salivary and lacrimal glands, leading to clinical symptoms of xerostomia and xerophthalmia. However, between 0.5% and 40% of patients may experience extraglandular manifestations, including renal, pulmonary, hematological, or neurological involvement. Additionally, up to 5% of individuals with SjD may develop lymphoma over the course of their disease [1].

TAFRO syndrome is a rare systemic inflammatory disorder first described in 2010 by Takai et al. [2]. It is characterized by thrombocytopenia, anasarca, fever, reticulin fibrosis, renal dysfunction, and organomegaly [3–5]. Although initially considered a variant of idiopathic multicentric Castleman disease (iMCD-TAFRO), emerging evidence suggests that TAFRO syndrome may represent a distinct hyperinflammatory condition with an autoimmune basis, driven by excessive production of cytokines, including interleukin-6 (IL-6) and vascular endothelial growth factor (VEGF) [5, 6]. Importantly, well-defined differential features have been described that distinguish TAFRO syndrome from iMCD not otherwise specified (iMCD-NOS) (Table 1) [7, 8].

**Table 1:** Differences between TAFRO and multicentric Castleman disease not otherwise specified (NOS)

Domain	TAFRO (iMCD-TAFRO)	iMCD-NOS
Typical clinical course	• Acute or subacute, aggressive, often rapidly progressive; many patients require hospitalization or ICU care	• More heterogeneous; may be subacute or chronic, with fluctuating inflammatory activity
Key clinical phenotype	• Anasarca is very prominent and characteristic • Fever • Renal dysfunction: common and often severe; acute kidney injury is typical • Organomegaly • Lymphadenopathy is often mild or even subtle, disproportionate to disease severity	• Constitutional symptoms • Hepatosplenomegaly • Renal involvement can occur but is less constant and usually milder • Lymphadenopathy is usually prominent, multicentric, and clinically evident
Hematologic profile	• Thrombocytopenia is a defining feature • Anemia of inflammation is common	• Thrombocytosis or normal platelet count
Hepatic profile	• Elevated serum ALP level, normal ALT and AST	• May be present but not defining
Inflammatory markers	• Lower level of serum gamma globulin • Elevated CRP and cytokine activation • Higher neutrophil counts • Predominant VEGF elevation • IL-6 is elevated but relatively less prominent	• Higher levels of serum gamma globulin • Higher increase in serum levels of IL-6 • VEGF normal or moderately increased, likely secondary
Bone marrow	• Reticulin fibrosis is characteristic • Megakaryocytic hyperplasia with slight atypia	• Bone marrow involvement may occur, but reticulin fibrosis is not typical or required
Lymph node	• Slightly enlarged lymph nodes in size: dense endothelial venules, large nuclei of endothelial cells, and expanded FDC network • Expanded interfollicular area, HHV-8 negative, no light chain restriction	• Sheets of plasma cells, hyperplastic germinal center • Expanded interfollicular area, HHV-8 negative, no light chain restriction
Treatment response	• Frequently refractory to corticosteroids alone often requires early combination immunosuppressive therapy (e.g., calcineurin inhibitors and/or anti-IL-6 therapy)	• Often responsive to anti-IL-6 therapy (siltuximab or tocilizumab), with or without corticosteroids

Information adapted from references 7 and 8

*ICU* intensive care unit, *ALP* alkaline phosphatase, *ALT* alanine aminotransferase, *AST* aspartate aminotransferase, *CRP* C-reactive protein, *VEGF* vascular endothelial growth factor, *IL-6* interleukin-6, *FDC* follicular dendritic cells, *HHV-8* human herpesvirus 8

The coexistence of SjD and TAFRO syndrome has been reported in isolated cases and recent reviews. These reports suggest a shared pathogenic mechanism that may involve IL-6, VEGF, and anti-SSA/Ro60 autoantibodies [6, 9, 10]. Early recognition of the association between these two conditions is crucial given the rapid progression of TAFRO syndrome and the potential benefits of targeted immunomodulatory therapies.

## Case report

A 33-year-old previously healthy woman presented to her local hospital with a 1-month history of diarrhea, subjective fever, anasarca, oliguria, cervical and axillary lymphadenopathy, and hepatosplenomegaly. Initial studies revealed non-hypertensive ascites (serum albumin/ascitic fluid albumin gradient: 0.7), severe thrombocytopenia (28 × 10^3^/uL, reference range (RR): 150–450 × 10^3^/uL), KDIGO (Kidney Disease Improving Global Outcomes) stage 3 acute kidney injury (AKI) with creatinine of 3.2 mg/dL (RR: 0.5–1.1 mg/dL), significant 24-h proteinuria (524 mg/2000 mL), ADAMTS 13: 16.9%, hemoglobin: 7.5 mg/dL, LDH: 580 U/L (RR: 120–246 U/L), and haptoglobin: 11.8 mg/dL (RR: 30–200 mg/dL).

She required management in the intensive care unit, including hemodynamic and ventilatory support, as well as renal replacement therapy (RRT). Systemic lupus erythematosus (SLE) was initially suspected based on the presence of polyserositis (ascites and pleural effusion) and a renal biopsy showing membranoproliferative glomerulonephritis (MPGN) with a thrombotic microangiopathy (TMA) pattern.

The patient received pulses of methylprednisolone (500 mg/day) for 3 days, and RRT was discontinued following clinical improvement in December 2024. She was discharged on high-dose prednisolone (60 mg/day) with a tapering plan, along with hydroxychloroquine 200 mg/day.

**Fig. 1:**
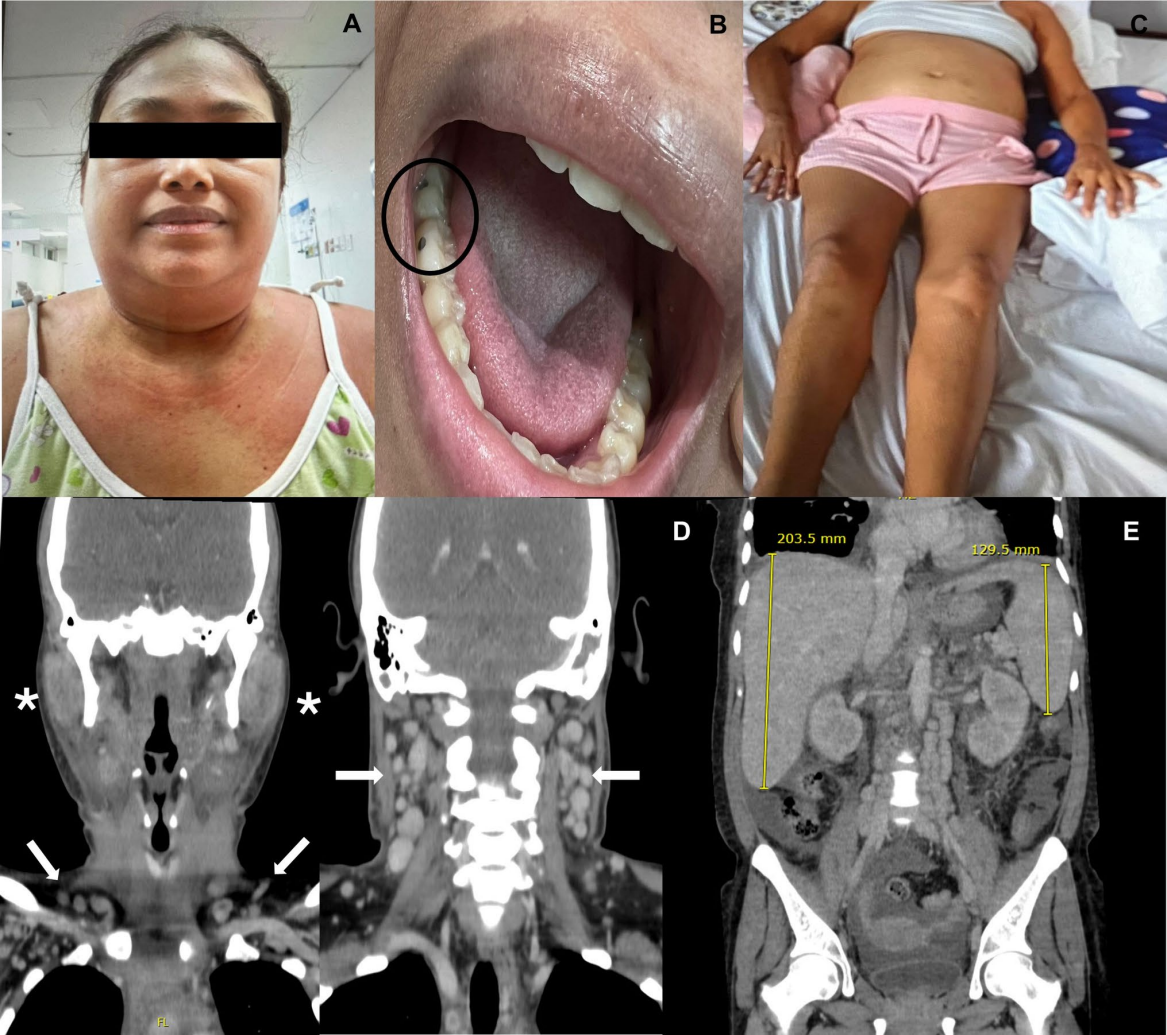
**A** Bilateral enlargement of the parotid glands. **B** Dental caries in atypical distribution areas (circle).** C** Anasarca. **D** Contrast-enhanced CT scan of the head and neck showing bilateral parotid gland enlargement (asterisk) and lymph nodes larger than 2 cm in the submandibular, cervical, and supraclavicular chains (arrows).** E** Contrast-enhanced CT scan of the abdomen revealing hepatosplenomegaly

For the next 7 months, the patient remained stable and adhered to treatment. However, the gradual reduction in glucocorticoid dosage was followed by a recurrence of symptoms, and she was admitted to our institution due to the recurrent anasarca and fever, along with fatigue, dry mouth, dry eyes, wrist arthralgia, and dyspnea on minimal exertion. Physical examination revealed a positive bilateral Schirmer test (< 5 mm/5 min), bilateral parotid enlargement, dental caries in atypical distribution, firm enlarged lymph nodes larger than 2 cm in the submandibular, cervical, and supraclavicular chains, and hepatosplenomegaly, which was better characterized by imaging studies (Fig. 1).

**Fig. 2:**
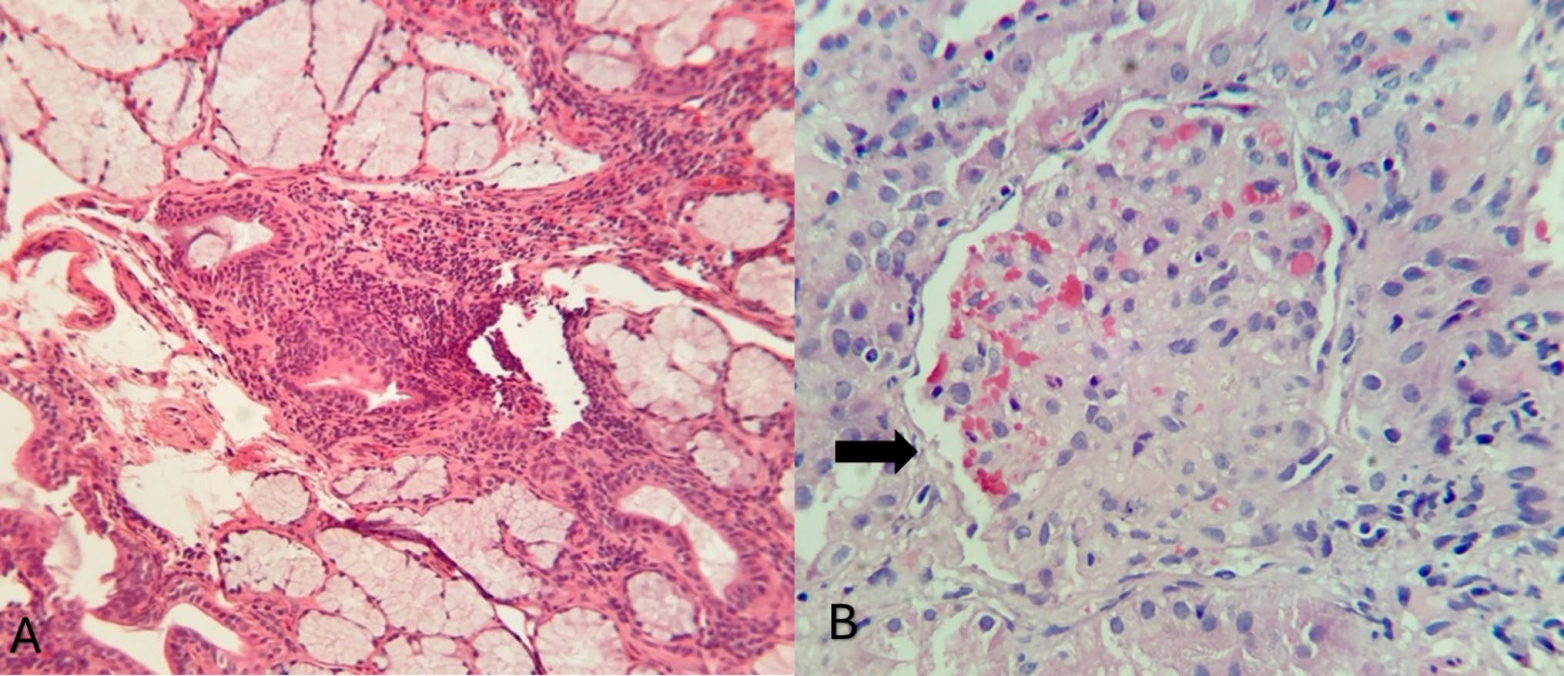
**A **Minor salivary gland biopsy showing focal periductal lymphocytic sialadenitis, adjacent to mucosal acini with normal appearance (hematoxylin and eosin (H&E), × 200). **B **Renal biopsy showing a glomerulus with a solidified and bloodless appearance, characterized by thickened capillary walls and occlusion of the capillary lumens. A segment with capillary congestion and erythrocyte fragmentation is also observed (arrow) (H&E, × 400)

All microbiological studies were negative. The immunoserological profile showed ANA at 1:320 with a fine granular nuclear pattern (AC-4), normal complement levels (C3: 158 mg/dL; C4: 31 mg/dL), and negative anti-dsDNA, anti-Sm, anti-RNP, and anti-La antibodies, with anti-Ro > 200 (positive). A minor salivary gland biopsy revealed focal lymphocytic sialadenitis with a focus score of 1.5 (Fig. 2A). Consequently, SLE was excluded, and the patient met the 2016 ACR/EULAR classification criteria for SjD (6 points) [1].

**Fig. 3:**
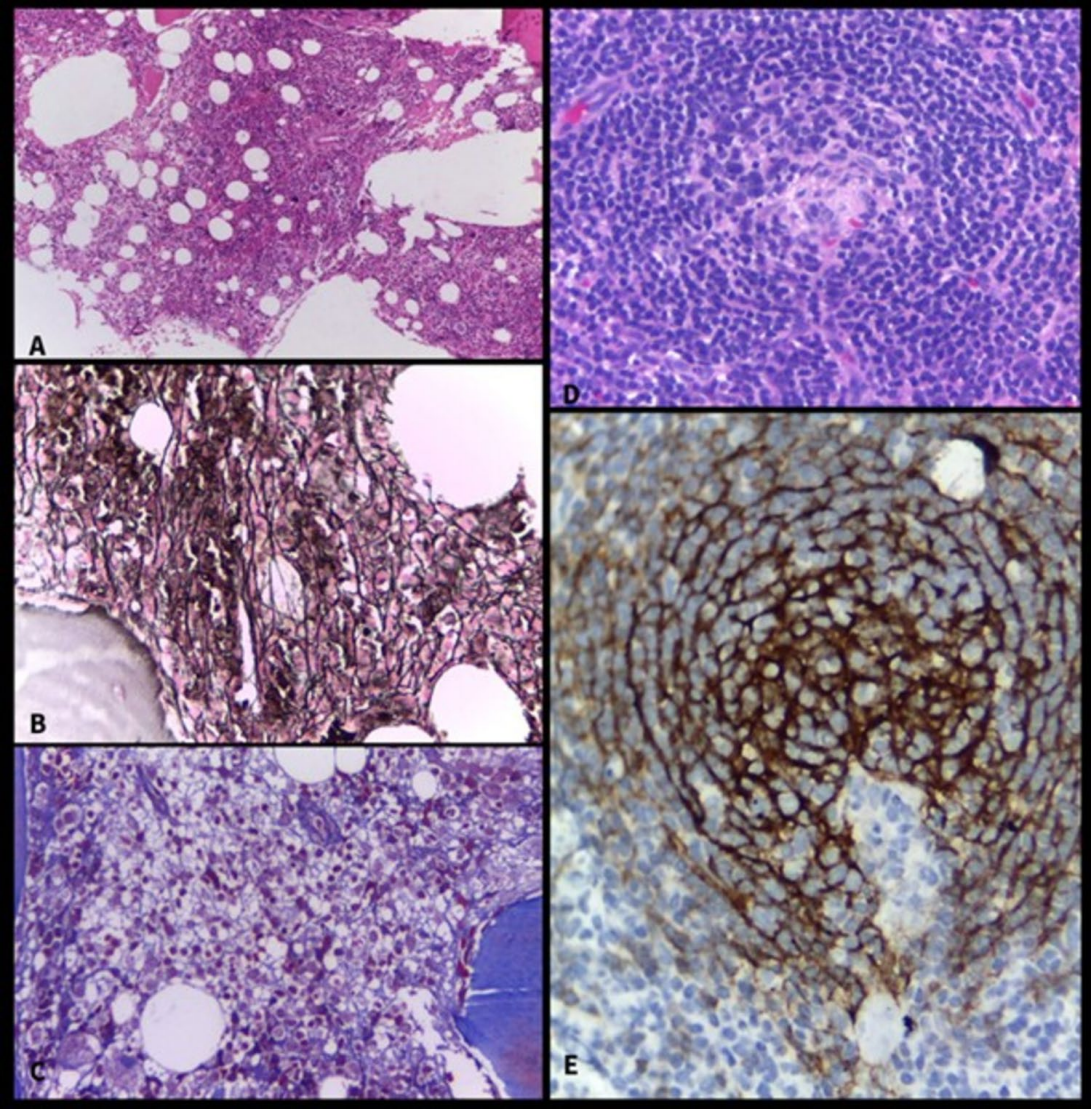
**A** Bone marrow stained with H&E, × 10 magnification, showing the presence of all three hematopoietic cell lines, an increased number of dystrophic megakaryocytes. **B** Reticulin stain demonstrating grade 3 bone marrow fibrosis. **C** Masson’s trichrome stain showing collagen fibrosis. **D** Lymph node stained with H&E, × 40 magnification, showing a lymphoid follicle with a hyaline-vascular lesion characterized by a regressed germinal center and a prominent radial blood vessel. **E** CD21 stain, × 40 magnification

A lymph node biopsy showed changes consistent with mixed hyaline vascular and plasma cell variant Castleman disease, and bone marrow studies revealed reticulin fibrosis (Fig. 3). The patient did not meet criteria for POEMS syndrome, and human herpesvirus 8 (HHV-8) testing was negative. And after applying the diagnostic criteria proposed by Masaki et al. [3], the patient fulfilled all three major criteria: anasarca, fever, and severe thrombocytopenia (30,000 × 10^3^/uL), as well as multiple minor criteria, including Castleman disease–like features on lymph node biopsy, reticulin myelofibrosis, hepatosplenomegaly, and progression to renal failure.

**Fig. 4:**
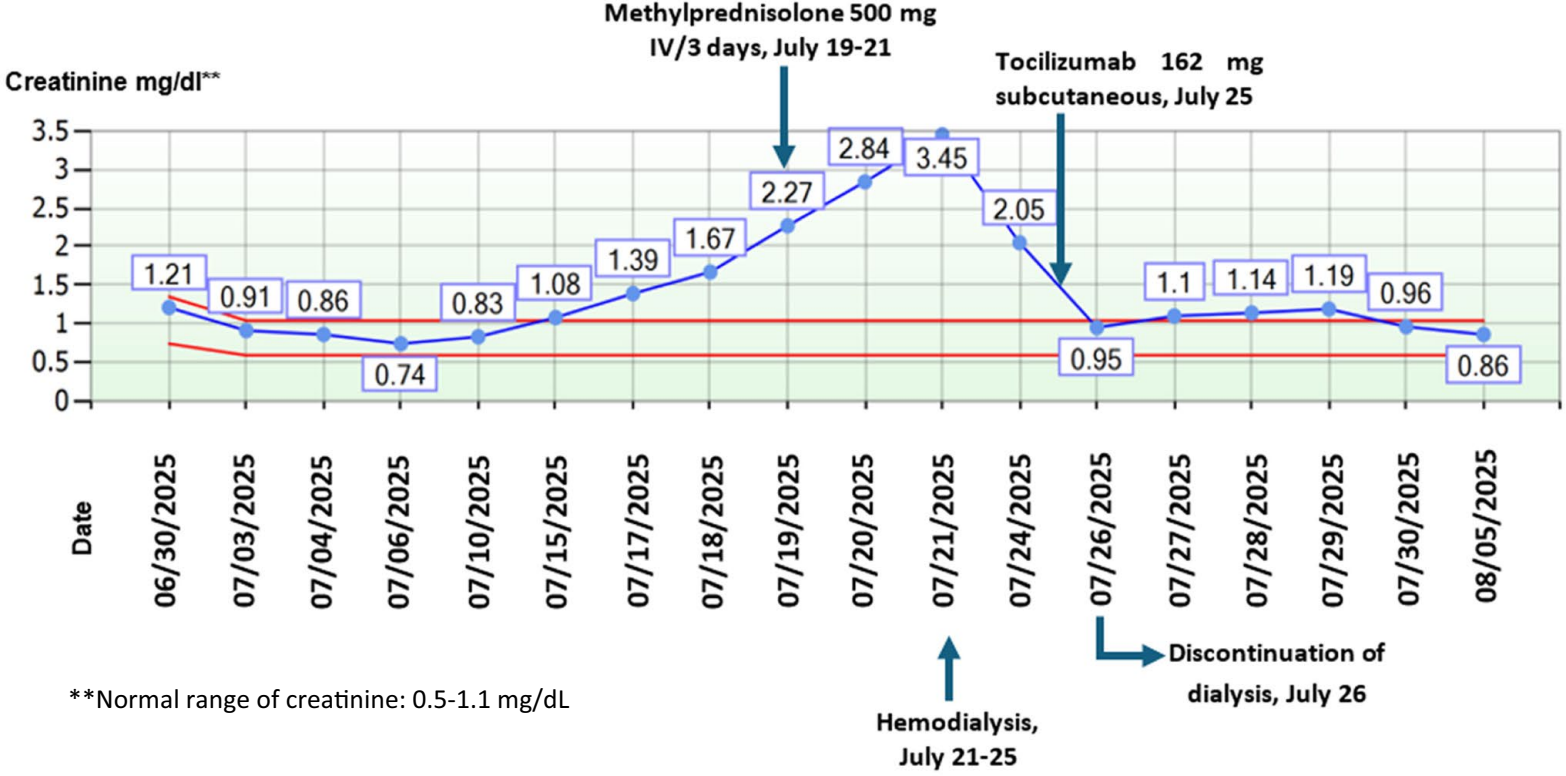
Evolution of renal function (creatinine) during the second hospitalization following methylprednisolone pulse therapy and subsequent treatment with tocilizumab

Treatment was initiated with prednisolone at a dose of 60 mg/day. Subsequently, she developed renal deterioration again meeting criteria for KDIGO stage 3 AKI (creatinine 2.27 mg/dL (RR: 0.5–1.1 mg/dL)), accompanied by acute pulmonary edema, anuria, and metabolic acidosis. Despite methylprednisolone pulses, there was no favorable response, necessitating initiation of RRT (July 21). Given the torpid clinical course, a repeat renal biopsy was performed to obtain a new histological evaluation aimed at once again excluding the presence of glomerular immune complex deposits and identifying additional lesions potentially responsible for renal deterioration, such as acute tubular necrosis or tubulointerstitial inflammation, as well as to assess disease progression and the degree of chronicity. The renal biopsy showed an MPGN pattern, characterized by double contours of the glomerular capillary walls, tubulointerstitial nephritis, and chronic TMA. Immunofluorescence studies in both biopsies showed no immune complex or complement deposits, thereby excluding immune complex–mediated MPGN and strongly supporting that the observed MPGN pattern was secondary to TMA (Fig. 2B). Given this clinical course, TAFRO syndrome associated with a systemic inflammatory response was considered. In-hospital treatment with tocilizumab 162 mg subcutaneously (SC) weekly for 8 weeks was initiated, due to the unavailability of the intravenous formulation, resulting in rapid clinical improvement, reversal of organ dysfunction, platelets 112,000 × 10^3^/uL and creatine 0.86 mg/dL, and progressive recovery of renal function, allowing discontinuation of dialysis in July 26 (Fig. 4). The patient was discharged to continue outpatient management with the IL-6 inhibitor but did not receive it due to insurance difficulties. During a follow-up evaluation, the patient experienced multiple episodes of vomiting accompanied by syncope and hypotension. Hospitalization was deemed necessary during a subsequent 20-day stay, and SC tocilizumab was resumed, resulting in improvement of her symptoms and subsequent discharge. At a follow-up appointment 15 days later, she was asymptomatic, and her laboratory values had normalized, so she continued with a prednisolone tapering regimen, at that time being at 40 mg/day and tocilizumab 162 mg SC weekly.

## Discussion

SjD is a systemic autoimmune disorder characterized by lymphocytic infiltration of the exocrine glands and by extraglandular manifestations of varying severity. In recent years, the coexistence of SjD with TAFRO syndrome (SjD-TAFRO) has been increasingly recognized. TAFRO is a hyperinflammatory condition characterized by thrombocytopenia, anasarca, fever, reticulin fibrosis, renal dysfunction, and organomegaly. Although initially considered a variant of iMCD, current evidence suggests that TAFRO represents an autonomous inflammatory syndrome driven by aberrant cytokine activation and with possible differential autoimmune bases [3–5].

In our case, the patient presented with fever, anasarca, severe thrombocytopenia, medullary fibrosis, and nephropathy secondary to thrombotic microangiopathy, meeting the major and minor criteria proposed by Masaki et al. (2019) and later validated internationally by Nishimura et al. [3, 4]. A systematic review by Grange et al. identified 10 published cases through 2022, of which 80% had a concomitant diagnosis of SjD, and 75% demonstrated histological findings compatible with Castleman’s disease. Among these cases, 90% were positive for anti-SSA antibodies and exhibited early renal impairment, supporting the hypothesis of a shared pathogenic mechanism [9]. Other reports have described recurrences or atypical manifestations, such as retroperitoneal hemorrhage in patients with SjD-TAFRO, further highlighting the clinical heterogeneity of this overlap syndrome [11].

Recent work by Shirakashi et al. has provided important insights into the immunological background of TAFRO syndrome, demonstrating that anti-SSA/Ro60 antibodies were present in up to 86% of patients with TAFRO, including individuals without clinical or histopathological evidence of SjD, whereas they were absent in patients with iMCD. These findings led the authors to propose that TAFRO syndrome may represent a distinct clinical entity within an anti-SSA antibody–associated immune spectrum, characterized by marked hypercytokinemia, endothelial dysfunction, and interferon-mediated immune activation. In this context, anti-SSA antibodies may not represent an incidental finding, but rather a biomarker of a specific immunopathogenic pathway underlying TAFRO syndrome [6]. Our case strongly supports this hypothesis, as the patient not only exhibited high anti-SSA/Ro60 titers but also fulfilled the 2016 ACR/EULAR classification criteria for Sjögren’s disease [1], suggesting that overt systemic autoimmunity and TAFRO syndrome may coexist and share common pathogenic mechanisms.

Watanabe et al. also observed that combining B-cell-directed immunomodulation with rituximab and tocilizumab achieved sustained remissions in refractory SjD-TAFRO cases, supporting the dual role of IL-6 signaling and B-cell hyperactivity in its pathophysiology [12].

From a pathophysiological perspective, SjD and the TAFRO syndrome share activation of convergent inflammatory pathways centered on IL-6, VEGF, BAFF, type I and II interferons, and JAK-STAT3/mTOR signaling [13, 14]. In SjD, anti-SSA autoantibodies induce epithelial damage and promote the release of type I interferons. In contrast, in TAFRO syndrome, a cytokine storm dominated by IL-6 and VEGF is responsible for massive edema and renal failure [15–17].

The identification of mutations in MEK2 (P128L) and RUNX1 (G60C) in iMCD-TAFRO supports the existence of an inflammatory clonal component that amplifies IL-3 and MAPK-ERK-mediated responses [18]. In parallel, Butzmann et al. and Sumiyoshi et al. reported epigenetic alterations in PDGFRB and NCOA4 that increase IL-6-dependent signalization, further consolidating a model of chronic endothelial inflammation and fibrosis [19, 20].

Treatment of SjD-TAFRO syndrome requires intensive immunosuppression. Although glucocorticoids are the initial basis of management, their efficacy as monotherapy is limited (less than 10%) [12]. Current evidence supports combining glucocorticoids with IL-6 inhibitors (tocilizumab or siltuximab) and, in some cases, calcineurin inhibitors such as ciclosporine or tacrolimus, which modulate T-cell-dependent activation [21]. In refractory disease, JAK1/2 inhibitors (ruxolitinib) and B-cell-directed therapies (rituximab) have been used, resulting in complete clinical responses [21, 22]. In our patient, the significant improvement after weekly tocilizumab supports the central role of the IL-6/VEGF axis in mediating hypercytokinemia.

## Conclusions

The overlap between SjD and TAFRO syndrome is an uncommon but potentially serious form of hyperinflammatory autoimmunity associated with high mortality. This case highlights the importance of early recognition of concurrent TAFRO and SjD in patients presenting with atypical features such as anasarca, cytopenias, and rapid progressive acute kidney dysfunction. Timely identification allows the implementation of targeted therapeutic strategies, such as initiating therapies, particularly IL-6 inhibition, which can be crucial for reversing the clinical course of the disease and improving survival.

## References

[1] Shiboski CH, Shiboski SC, Seror R, Criswell LA, Labetoulle M, Lietman TM, Rasmussen A, Scofield H, Vitali C, Bowman SJ, Mariette X, International Sjögren’s Syndrome Criteria Working Group (2017) 2016 American College of Rheumatology/European League against rheumatism classification criteria for primary Sjögren’s syndrome: a consensus and data-driven methodology involving three international patient cohorts. Arthritis Rheumatol 69(1):35–45. 10.1002/art.39859 27785888 10.1002/art.39859 PMC5650478

[2] Takai K, Nikkuni K, Shibuya H, Hashidate H (2010) Thrombocytopenia with mild bone marrow fibrosis accompanied by fever, pleural effusion, ascites and hepatosplenomegaly. Rinsho Ketsueki 51(5):320–325 20534952

[3] Nishimura Y, Fajgenbaum DC, Pierson SK, Iwaki N, Nishikori A, Kawano M, Nakamura N, Izutsu K, Takeuchi K, Nishimura MF, Maeda Y, Otsuka F, Yoshizaki K, Oksenhendler E, van Rhee F, Sato Y (2021) Validated international definition of the thrombocytopenia, anasarca, fever, reticulin fibrosis, renal insufficiency, and organomegaly clinical subtype (TAFRO) of idiopathic multicentric Castleman disease. Am J Hematol 96(10):1241–1252. 10.1002/ajh.26292 34265103 10.1002/ajh.26292 PMC9642098

[4] Masaki Y, Kawabata H, Takai K, Tsukamoto N, Fujimoto S, Ishigaki Y, Japanese TAFRO Syndrome Research Team et al (2020) 2019 updated diagnostic criteria and disease severity classification for TAFRO syndrome. Int J Hematol 111(1):155–8. 10.1007/s12185-019-02780-1 10.1007/s12185-019-02780-1 31782045

[5] Masaki Y, Arita K, Sakai T, Takai K, Aoki S, Kawabata H (2022) Castleman disease and TAFRO syndrome. Ann Hematol 101(2):485–490. 10.1007/s00277-022-04762-6 35044513 10.1007/s00277-022-04762-6 PMC8768434

[6] Shirakashi M, Nishida Y, Nakashima R, Fujimoto M, Hiwa R, Tsuji H, Kitagori K, Akizuki S, Morinobu A, Yoshifuji H (2024) TAFRO syndrome is associated with anti-SSA/Ro60 antibodies, in contrast to idiopathic castleman disease. Sci Rep 14(1):2889. 10.1038/s41598-024-53413-5 38311632 10.1038/s41598-024-53413-5 PMC10838910

[7] Iwaki N, Fajgenbaum DC, Nabel CS, Gion Y, Kondo E, Kawano M, Masunari T, Yoshida I, Moro H, Nikkuni K, Takai K, Matsue K, Kurosawa M, Hagihara M, Saito A, Okamoto M, Yokota K, Hiraiwa S, Nakamura N, Nakao S, Yoshino T, Sato Y (2016) Clinicopathologic analysis of TAFRO syndrome demonstrates a distinct subtype of HHV-8-negative multicentric Castleman disease. Am J Hematol 91(2):220–226. 10.1002/ajh.24242 26805758 10.1002/ajh.24242

[8] Fajgenbaum DC, Uldrick TS, Bagg A, Frank D, Wu D, Srkalovic G, Simpson D, Liu AY, Menke D, Chandrakasan S, Lechowicz MJ, Wong RS, Pierson S, Paessler M, Rossi JF, Ide M, Ruth J, Croglio M, Suarez A, Krymskaya V, Chadburn A, Colleoni G, Nasta S, Jayanthan R, Nabel CS, Casper C, Dispenzieri A, Fosså A, Kelleher D, Kurzrock R, Voorhees P, Dogan A, Yoshizaki K, van Rhee F, Oksenhendler E, Jaffe ES, Elenitoba-Johnson KS, Lim MS (2017) International, evidence-based consensus diagnostic criteria for HHV-8-negative/idiopathic multicentric Castleman disease. Blood 129(12):1646–1657. 10.1182/blood-2016-10-746933 28087540 10.1182/blood-2016-10-746933 PMC5364342

[9] Grange L, Chalayer E, Boutboul D, Paul S, Galicier L, Gramont B, Killian M (2022) TAFRO syndrome: a severe manifestation of Sjogren’s syndrome? A systematic review. Autoimmun Rev 21(8):103137. 10.1016/j.autrev.2022.103137 35803499 10.1016/j.autrev.2022.103137

[10] Takai K (2024) TAFRO syndrome: a syndrome or a subtype of multicentric Castleman disease? Biomedicines 12(3):652. 10.3390/biomedicines12030652 38540266 10.3390/biomedicines12030652 PMC10968353

[11] Ohta T, Oda N, Saito K, Tamiya S, Ueno T (2020) A case of repeated TAFRO syndrome-like symptoms and retroperitoneal hemorrhage in a patient with Sjögren syndrome. Cureus 12(12):e12175. 10.7759/cureus.12175 33489586 10.7759/cureus.12175 PMC7813549

[12] Watanabe M, Takahashi H, Iwasaki H, Kanda J, Ito T, Matsumoto K et al (2023) Combined B-cell immunomodulation with rituximab and IL-6 blockade with tocilizumab in refractory TAFRO syndrome. Front Immunol 14:1528465. 10.3389/fimmu.2023.1528465

[13] Tu KH, Fan PY, Chen TD, Chuang WY, Wu CY, Ku CL, Tian YC, Yang CW, Fang JT, Yang HY (2021) TAFRO syndrome with renal thrombotic microangiopathy: insights into the molecular mechanism and treatment opportunities. Int J Mol Sci 22(12):6286. 10.3390/ijms22126286 34208103 10.3390/ijms22126286 PMC8230834

[14] Miura K, Nishimaki-Watanabe H, Takahashi H, Nakagawa M, Otake S, Hamada T, Koike T, Iizuka K, Takeuchi Y, Kurihara K, Endo T, Ito S, Nukariya H, Namiki T, Hayashi Y, Nakamura H (2024) TAFRO syndrome: guidance for managing patients presenting thrombocytopenia, anasarca, fever, reticulin fibrosis, renal insufficiency, and organomegaly. Biomedicines 12(6):1277. 10.3390/biomedicines12061277 38927484 10.3390/biomedicines12061277 PMC11200895

[15] Kakutani T, Kamada R, Tamai Y (2024) Pathophysiology, treatment, and prognosis of thrombocytopenia, anasarca, fever, reticulin fibrosis/renal failure, and organomegaly (TAFRO) syndrome: a review. Curr Issues Mol Biol 46(10):11255–11269. 10.3390/cimb46100668 39451548 10.3390/cimb46100668 PMC11506032

[16] Hasegawa E, Okada M, Ozawa S, Takada M et al (2021) TAFRO syndrome associated with systemic lupus erythematosus: a case report and literature review. Int J Hematol 113(2):281–287. 10.1007/s12185-020-03078-8

[17] Li ZY, Kim S, Huang S, Mian R (2019) Multicentric castleman disease with TAFRO syndrome and Sjögren’s. Clin Case Rep 7(12):2388–2392. 10.1002/ccr3.2502 31893065 10.1002/ccr3.2502 PMC6935625

[18] Yoshimi A, Trippett TM, Zhang N, Chen X, Penson AV, Arcila ME, Pichardo J, Baik J, Sigler A, Harada H, Fajgenbaum DC, Dogan A, Abdel-Wahab O, Xiao W (2020) Genetic basis for iMCD-TAFRO. Oncogene 39(15):3218–3225. 10.1038/s41388-020-1204-9 10.1038/s41388-020-1204-9 PMC7148173 32051554

[19] Butzmann A, Kumar J, Sridhar K, Gollapudi S, Ohgami RS (2021) A review of genetic abnormalities in unicentric and multicentric castleman disease. Biology 10(4):251. 10.3390/biology10040251 33804823 10.3390/biology10040251 PMC8063830

[20] Sumiyoshi R, Koga T, Kawakami A (2024) Biomarkers and signaling pathways implicated in the pathogenesis of idiopathic multicentric castleman disease/thrombocytopenia, anasarca, fever, reticulin fibrosis, renal insufficiency, and organomegaly (TAFRO) syndrome. Biomedicines 12(6):1141. 10.3390/biomedicines12061141 38927348 10.3390/biomedicines12061141 PMC11200392

[21] Killian M, Viel S, Chalayer E, Forest F, Grange S, Bonnefoy PB, Oksenhendler E, Cathébras P, Paul S (2021) JAK1/2 inhibition in severe TAFRO syndrome: a case report. Ann Intern Med 174(5):717–720. 10.7326/L20-1051 33428440 10.7326/L20-1051

[22] Masuda T, Suzuki T, Ohshima M, Suzuki A, Minemura N, Nakajima H (2025) TAFRO-like symptoms in a Sjögren’s syndrome patient with HTLV-1 infection. Intern Med 64(5):1107–1112. 10.2169/internalmedicine.4124-24 39198166 10.2169/internalmedicine.4124-24 PMC12021513

